# Antiplasmodial Activity of Methylangolensate From *Entandrophragma angolense* (Welw.) C.DC. Stem Bark: An In Vitro and In Silico Approach

**DOI:** 10.1155/jotm/7224187

**Published:** 2026-09-11

**Authors:** Jude Tetteh, Jemima Aggrey Appiah, Felix Zoiku, Derwaa Keteku, Bismark Aboagye-Adjei, Arnold Donkor Forkuo, Isaac Kingsley Amponsah, Charles Ansah, Kwesi Boadu Mensah

**Affiliations:** ^1^ Department of Pharmacology, Kwame Nkrumah University of Science and Technology, Kumasi, Ghana, knust.edu.gh; ^2^ Department of Pharmaceutical Science, Kumasi Technical University, Kumasi, Ghana, kstu.edu.gh; ^3^ Noguchi Memorial Institute for Medical Research, University of Ghana, Accra, Ghana, ug.edu.gh

**Keywords:** drug likeness, methylangolensate, molecular docking, molecular dynamic simulations, multistage

## Abstract

**Background:**

Previous investigation of the antiplasmodial effects of methylangolensate (MAL) has revealed promising activity against multiple strains of *Plasmodium falciparum* parasites. However, a significant gap still remains to be bridged regarding its potential as a lead compound for the development of novel drugs for the treatment of malaria. The study seeks to evaluate the multistage antiplasmodial effects, drug likeness, in silico ADMET properties, and possible drug targets of MAL against the *P. falciparum* parasites.

**Methods:**

The cytotoxic effects of MAL on HepG2 cells and the inhibitory effects of MAL against the blood stages of both 3D7 *P. falciparum* and Dd2 *P. falciparum* were assessed using a tetrazolium‐based colorimetric test and SYBR Green assay, respectively. The nature of the interactions between MAL and some validated drug targets of malaria (DHFR‐TS, *Pf*LDH, FP‐2, plasmepsins II, *Pf*DHODH and *Pf*HGXPRT) were determined using molecular docking and molecular dynamics simulations. The drug likeness of MAL was predicted using rule‐based filters such as Lipinski. The ADMET properties of MAL were predicted using the SwissADME online tool. A modified in vitro antiplasmodial method involving the supplementation of the culture media with varying concentrations of folic acid was used to validate the antifolate potential of MAL.

**Results:**

MAL demonstrated minimal cytotoxic effects (CC_50_ of 89.39 μg/mL) against HepG2 liver cells. MAL demonstrated a good antiplasmodial effect against the trophozoites and schizonts (IC_50_ of 0.8568–5.1210 μg/mL) and moderate gametocytocidal activity (IC_50_ of 12.0800–18.8200 μg/mL) against both 3D7 *P. falciparum* and Dd2 *P. falciparum* parasites. MAL met all the criteria set by Lipinski, Ghose, Veber, and Abbott bioavailability score for a drug‐like compound and had favorable predicted ADMET properties. The compound demonstrated a strong affinity for the active sites of DHFR‐TS, *Pf*LDH main pocket, *Pf*LDH cofactor active site, falcipain‐2, plasmepsins II, *Pf*DHODH, and *Pf*HGXPRT with binding energies of −10.54, −7.25, −8.05, −7.77, −7.39, −9.41, and −8.02 Kcal/mol, respectively. Molecular dynamics simulation revealed stable docked MAL–protein complexes. MAL showed good potential as an antifolate agent.

**Conclusion:**

MAL may serve as a lead compound for the design and development of novel drugs that elicit their antiplasmodial effects by interfering with folate metabolism, DNA synthesis, energy metabolism, and nutrition of the *P. falciparum* parasites.

## 1. Background

Malaria continues to pose serious threat to global health despite the various strategies by the World Health Organizations (WHO) for eradication. Malaria cases increased by 9 million from 2023 to 2024, with the WHO African Region accounting for 94% of the cases [1]. Globally, malaria‐associated mortalities have increased from 578,000 deaths in 2015 to 610,000 deaths in 2024 [1]. Only four African countries (Nigeria, Tanzania, DR. Congo, and Niger) were plagued by 50% of these deaths, which were mostly among young children [1].

Arguably, the greatest menace to the fight against malaria is the emergence of resistance by the *Plasmodium falciparum* parasites to the first‐line antimalarial therapies. There is an urgent need for newer drugs to replace the conventional antimalarial drugs before an epidemic of drug‐resistant malaria develops. Plants and their isolated phytochemicals have contributed immensely to the treatment of malaria. Quinolines and artemisinins, which are the mainstay for the management of malaria, were derived from plants. Actually, many drugs currently used for the treatment of malaria are alkaloids or their synthetic derivatives [2]. Many herbs and phytochemicals have been investigated for their activity against various strains of the plasmodium parasites in both in vivo and in vitro antiplasmodial assays [2]. A systematic review by Tajabakhsh et al. on the antiplasmodial activity and toxicity of African medicinal plants identified 722 research works on malaria involving 502 plants species [3]. The study identified isolated phytochemicals from these plants to demonstrate better antiplasmodial activity compared to their crude extracts (*p* value = 0.044) [3]. Hence, there is the need to expedite research works toward identifying novel phytochemicals for the treatment of malaria.

*Entandrophragma angolense* (Welw.) C.DC. is a plant species native to tropical Africa. It is distributed from Guinea to Angola East and to Kenya and Tanzania. It is mostly found in moist deciduous forest and sometimes in the Evergreen‐forest. It occurs in lowlands and mid‐altitude rainforests, but sometimes also in gallery forests and thickets [4]. The plant is used traditionally in the treatment of fever‐related conditions including malaria. The traditional use of *E*. *angolense* (Welw.) C.DC. as an antimalarial has been well established in the literature. The plant had been reported to be active against different lab strains of *P. falciparum* in vitro and against *P. berghei* in vivo [5, 6]. *E*. *angolense* (Welw.) C.DC. has also demonstrated good antiplasmodial effect against clinical isolates from Ivory Coast [6]. Some studies had investigated the antiplasmodial effects of certain phytochemicals isolated from *E*. *angolense* (Welw.) C.DC. and had identified limonoids including methylangolensate (MAL) to be responsible for its antimalarial property [7]. Limonoids have been reported by many studies to possess antiplasmodial properties. A study tested the antiplasmodial activity of 27 limonoids and found the limonoid gedunin to be the most active limonoid in vitro against *P. falciparum* (IC_50_ = 0.72 μg/mL), but unfortunately, gedunin was ineffective in vivo against *P*. *berghei* at a dose of 90 mg/kg/day [8]. Another study has reported on the improvement of the in vivo activity of gedunin against *P. berghei* through the correction of issues pertaining to formulations [9]. A study revealed comparable antiplasmodial activity between gedunin and quinine [10]. Bickii et al. also reported gedunin to be the most active among 1‐deacetylkhivorin, 7‐deacetylkhivorin, MAL, and 6‐acetylswietenolide against *P. falciparum* W2‐Indochina strain [11]. The IC_50_ of the limonoids ranged from 1.25 to 9.63 μg/mL [11]. The same study reported addictive interaction between gedunin and chloroquine [11]. In investigating the antiplasmodial activity of the limonoids, nimocinol, nimbandiol, meldenin, and isomeldenin, meldenin was found to be the most active limonoid (IC_50_ = 5.23 μg/mL) against the chloroquine‐resistant *P. falciparum* strain [12]. The limonoids, mahonin, 6α‐acetoxyepoxyazadiradione, azadiradione, epoxyazadiradione, and dysobin, were all found to be active against *P. falciparum* (IC_50_ of 2.06–6.31 μg/mL) in vitro. Domesticulides (B, C, and D), MAL, methyl 6‐acetoxyangolensate, azadiradione, dukunolide, and 6‐acetoxymexicanolide were also found to be active against *P. falciparum* in vitro with an IC_50_ of 2.4–9.7 μg/mL [12]. MAL is a limonoid that has been isolated and characterized from plants species of the *Khaya* and *Entandrophragma* genus. MAL has been reported to possess anti‐inflammatory, antimalarial, antiulcer, anticancer, and antifeedant activities. The investigation of the antiplasmodial activity of MAL has revealed promising effects against multiple strains of *P. falciparum* parasites (W2‐Indochina strain, chloroquine‐resistant Dd2 strain, and chloroquine‐sensitive 3D7 strain) in vitro [11, 13].

Malaria is a protozoan infection caused by *Plasmodium* species. The *P*. *falciparum* parasite is the deadliest species responsible for severe forms of the infection. *P. falciparum* has a complex life cycle that occurs in both humans and mosquitoes. The life cycle comprises of different forms or stages: asexual (blood and liver stage) and sexual (gametocytes). Current treatments do not inhibit the sexual and liver stages of infection; instead, they are only effective during the asexual blood stage [14]. Despite the fact that the clinical phase of malaria is caused by the asexual stage, it is crucial to create agents that target the sexual and hepatic stages of the parasites in order to prevent disease epidemics and protect vulnerable populations because asexual antimalarial medication resistance is on the rise [14]. All the stages of the parasites provide exploitable biochemical pathways that could be targeted by drug therapy [15]. The asexual stages (trophozoites and schizonts) are known to be more susceptible to drug therapy compared to the sexual stage (gametocytes) since they are more metabolically active [16]. For instance, the trophozoites actively digest host hemoglobin using proteases (plasmepsins and falcipains) to obtain amino acids necessary for growth and replication and therefore become susceptible to drugs that target these enzymes. Also, since the asexual parasites undergo extensive cell division, they become liable to drugs that interfere with enzymes such as dihydrofolate reductase‐thymidylate synthetase (DHFR‐TS) (folate metabolism), *P. falciparum* dihydroorotate dehydrogenase (*Pf*DHODH) (de novo biosynthesis of pyrimidines), and *P. falciparum* hypoxanthine–guanine–xanthine phosphoribosyltransferase (*Pf*HGXPRT) (purine salvage pathway), which are essential for DNA replication. When gametocytes develop, their total metabolic activity decreases until they attain maturity and quiescence. Only primaquine is clinically licensed to kill quiescent late‐stage gametocytes, despite the fact that various antimalarials, such as artemisinin derivatives, chloroquine, quinine, and atovaquone, have some efficacy against early stage gametocytes [16]. However, safer gametocyte‐targeted treatments are preferred because primaquine has some significant side effects. Despite the limited effect of conventional antimalarial drugs to the gametocytes, drug resistance has been linked to the gametocytes that carry the parasites from humans to mosquitoes [17]. Hence, to combat antimalarial resistance and eradicate malaria, it would be crucial that agents that possess multistage antiplasmodial effects are developed.

Finding novel substances having multistage antimalarial activity—that is, the ability to block hepatic, asexual, and sexual blood stages—is emphasized in the updated guidelines for the eradication of malaria [18]. This strategy is crucial since it is one of the most important ways to eradicate malaria. Therefore, to address the problem of growing resistance, active therapeutic molecules whose potency is not limited to a range of pathogenic asexual blood stages are required. Despite the promising antiplasmodial effects reported for MAL and the other limonoids, data are lacking in the literature on the exact stage of the malaria parasite they affect and which biochemical drug targets are responsible for their activity. Identification of the stage of the *P. falciparum* parasites MAL attacks and the biochemical pathway affected by the limonoid could help focus research attempts in the design and development of more potent analogs through structure‐based drug design. These analogs may become the new phase of antimalarial drug therapy. Hence, the aim of this study is to identify the stage specific antiplasmodial effects of MAL isolated from the stem bark of *E*. *angolense* (Welw.) C.DC. against both chloroquine‐sensitive 3D7 *P. falciparum* and chloroquine resistance Dd2 *P. falciparum* and to predict through molecular docking and molecular dynamic (MD) simulations, which biochemical drug targets might be exploited by MAL to kill the parasites. The study would also report on the drug likeness and ADMET properties of MAL using in silico techniques.

## 2. Materials and Methods

### 2.1. Source of MAL and Other Chemicals

The pure powder of methylangolensate (MAE) was donated by Prof. Isaac Kingsley Amponsah, Department of Pharmacognosy, Kwame Nkrumah University of Science and Technology, Kumasi—Ghana. The compound was isolated from a 70% ethanolic extract of the stem bark of *E*. *angolense* (Welw.) C.DC (Meliaceae family). The source of the plant material, the preparation of the 70% ethanolic extract of the stem bark of the plant, and the isolation and characterization of the MAL were as described by Sarpong et al. [19]. All other chemicals including artesunate (ART), RPMI, HEPES, and AlbuMAX were obtained from Sigma‐Aldrich.

### 2.2. Plasmodium Parasites and HepG2 Cells

The Noguchi Memorial Institute for Medical Research, University of Ghana, Legon, provided the chloroquine‐sensitive 3D7 and chloroquine‐resistant Dd2 lab strains of *P. falciparum parasites*. The HepG2 liver cells (ATCC HB‐8065) were obtained from the Department of Clinical pathology at Noguchi Memorial Institute for Medical Research. The Department of Clinical pathology at Noguchi Memorial Institute for Medical Research also received the HepG2 liver cells (ATCC HB‐8065) as a donation from Nagasaki University, Japan.

### 2.3. Preparation of Compounds for In Vitro Assays

#### 2.3.1. Preparation of MAL

A 1000 μg/mL solution of MAL in 0.5% DMSO was prepared from a 10 mg/mL stock solution. This solution was diluted 10‐fold to obtain a 100 μg/mL working solution. Subsequently, the 100 μg/mL solution was diluted ninefold to obtain nine separate assay solutions of MAL ranging from 100 to 0.39 μg/mL for plating.

#### 2.3.2. Preparation of ART

A 15 × 10^6^ ng/mL stock solution of ART was prepared by dissolving 0.075 g of ART in 5 mL of 5% DMSO. A 1:100 (10 μL in 990 μL) dilution of the stock solution was performed to obtain a 15 × 10^4^ ng/mL solution. A 1:100 (10 μL in 990 μL) dilution of this solution was done to obtain a 15 × 10^2^ ng/mL solution. A 1:10 (100 μL in 900 μL) dilution of the 15 × 10^2^ ng/mL solution was done to obtain a 150 ng/mL solution. This solution was further diluted (100 μL in 900 μL) to obtain a 15 ng/mL working solution. The 15 ng/mL solution was serially diluted ninefold to obtain the assay solution (15–0.01 ng/mL) for plating. All dilutions were made in 5% DMSO.

### 2.4. In Vitro Antiplasmodial Assay

#### 2.4.1. Cultivation of Parasites

##### 2.4.1.1. Cultivation of Trophozoites of *P. falciparum*

The trophozoites of both 3D7 and Dd2 lab strains of *P. falciparum* were cultured using the method described by Trager and Jensen, (1976) with some few modifications [20]. In this method, a continuous culture of asexual plasmodium parasites was maintained in a complete culture medium (10.44 g/L RPMI 1640, 5.94 g/L HEPES, 5 g/L AlbuMAX II, 50 mg/L hypoxanthine, 2.1 g/L sodium bicarbonate) at 37°C in an atmosphere of 93% N_2_, 4% CO_2_, and 3% O_2_ as described by Kumatia et al. [21]. In summary, the parasites were cultured in O + erythrocytes till a 5% parasitemia was attained. At this point, the culture was treated with 5% sorbitol to obtain synchronized trophozoites. A suspension of the synchronized trophozoites was prepared using uninfected blood and maintained in a complete culture medium (2% hematocrit with 1% parasitemia) for plating.

##### 2.4.1.2. Cultivation of Schizonts of *P. falciparum*

The schizonts of 3D7 and Dd2 lab strains of *P. falciparum* were cultured using the method as described by Trager and Jensen with some few modifications [20]. To ensure that the culture consists predominantly of schizonts, the parasites were cultured in O + erythrocytes until 8% parasitemia was determined through daily monitoring of Giemsa‐stained slides under the microscope. At 8% parasitemia, the culture was treated with 60% Percoll (30 mL Percoll + 17 mL RPMI + 3 mL 10XPBS) to obtain synchronized schizonts. The density gradient centrifugation using Percoll as described by Kumatia et al. [21] was then carried out to purify the synchronized schizonts. A suspension of the schizonts (2% hematocrit) was then prepared using fresh uninfected red cells and complete culture media for plating with compounds.

##### 2.4.1.3. Cultivation of Gametocytes of *P. falciparum*

The gametocytes of 3D7 and Dd2 lab strains of *P. falciparum* were cultured using the method as described by Trager and Jensen with some few modifications [20]. The method employed maintained a continuous culture of gametocytes in vitro in a complete medium (similar to that used for the trophozoites) as described by Kumatia et al. [21]. In summary, the parasites were cultured in O + erythrocytes in a T75 flask and culture monitored till 8%–10% parasitemia was obtained. At 8%–10% parasitemia, most of the parasites were in the trophozoite stage. To obtain synchronized trophozoites at this point, 5% sorbitol was added to the culture and afterward, the culture was treated with complete parasite media (CPM) and 50 mM N‐acetyl glucosamine (Day 0). A calm flow of gas was used to flush the flask and afterward kept in an incubator. From Day 1 through Day 4, spent media without erythrocytes were picked from the culture. On each day, CPM‐50 mM N‐acetyl glucosamine was added to the flask and then gassed before returning into the incubator. Spent media was again taken out from the culture on Day 5 but only CPM was added to the culture before incubation. The flask was gassed repeatedly for two weeks, and percentage gametocyte was determined from the number of gametocytes per every 1000 erythrocytes. A parasite suspension of gametocyte was now prepared for plating.

#### 2.4.2. Plating of MAL Against the Plasmodium Parasites

About 100 μL assay solutions of MAL (100–0.39 μg/mL) were plated in duplicate in a 96‐well coastal plate. A 15 ng/mL working solution of ART was diluted ninefold, and each concentration was plated side by side with MAL as a standard control. Approximately, 100 μL of parasite mix prepared was added to each well containing MAL and ART. Parasite mix was also added to wells containing only 0.5% DMSO (vehicle) to serve as negative control. The procedure was replicated for the MAL, ART and vehicle against trophozoites, schizonts, and gametocytes of both 3D7 *P. falciparum* and Dd2 *P. falciparum* parasites. The plates containing the MAL and ART plated against the various stages of the parasites were kept in a modular chamber. The plates were gassed with a mixture of gas as specified by Kumatia et al. [21] (93% N_2_, 4% CO_2_, and 3% O_2_) and kept at 37°C for 72 h.

#### 2.4.3. SYBR Green Assay for Evaluating the Antiplasmodial Effects of MAL

The SYBR Green assay was conducted as described by de Souza et al. with slight modifications [22]. To terminate the antiplasmodial assay, a buffer solution containing SYBR Green (100 μL) was added to the wells after 72 h of incubation to lyse the RBCs and to intercalate the DNA of the parasites. Afterward, the plates were kept away from light for about an hour. Fluorescence was then measured for each well at 470 and 520 nm using a fluorometer plate reader. MAL was tested in duplicate, and the concentrations that inhibited the trophozoites, schizonts, and gametocytes of both 3D7 *P. falciparum* and Dd2 *P. falciparum* by 50% (IC_50_) were estimated.

### 2.5. In Vitro Cytotoxicity Assay

The cytotoxic effect of MAL against HepG2 liver cells was investigated using the tetrazolium‐based calorimetric assay method as described by Kwansa‐bentum et al., with some modification [23]. The assay method is based on the ability of viable cells to enzymatically convert yellowish tetrazolium into dark purple formazan. Assay solutions of MAL in 5% DMSO ranging from 6.25 to 100 μg/mL were used. The HepG2 cells were cultivated in the DMEM culture medium, which was supplemented with fetal bovine serum (10%) and penicillin streptomycin L‐glutamine (1%) from Sigma‐Aldrich, Urbana, IL, USA. The HepG2 cells were maintained in a humified incubator gassed with 5% CO_2_ at 37°C and passaged upon attaining approximately 80% confluency. A 100 μL of the HepG2 cells containing about 1 × 10^5^ cells was seeded in a 96‐well microtiter plate. A 100 μL of each assay solution of MAL was added to the cells in the wells in triplicate, and the plates were kept in an incubator (5% CO2 at 37°C) for 72 h. Following incubation, phosphate‐buffered MTT solution was added to the wells and the plate was incubated for an additional 2 h. After 2 h, the culture medium (150 μL aliquots) was removed from each well and replaced by 200 μL of Triton X‐100 in acidified isopropanol to dissolve any formazan formed. Afterward, the plates were kept away from light for 24 h. The optical densities of the wells were ascertained using a plate reader at 570 nm. ART at the same concentrations as the assay solutions of MAL was plated alongside for comparison. CC_50_, which is the concentration at which 50% cytotoxicity occurred, was determined for both MAL and ART.

### 2.6. *In Silico* Assessment of the Potential Target Proteins Responsible for the Antiplasmodial Effects of MAL

#### 2.6.1. Molecular Docking

Molecular docking of MAL against some key target proteins of *P. falciparum* was carried out to determine the possible mechanism underlining the antiplasmodial effects of the compound. MAL was docked against six target proteins of *P. falciparum*; dihydrofolate reductase‐thymidylate synthase (*Pf*DHFR‐TS) (PDB ID: 4DP3), *P. falciparum* lactate dehydrogenase (*Pf*LDH) (PDB ID: 1LDG), falcipain‐2 (FP‐2) (PDB ID: 3BPF), plasmepsin II (PMS‐II) (PDB ID: 1LF3), *Pf*DHODH (PDB ID: 7KZ4) and *Pf*HGXPRT (PDB ID: 3OZF). DHFR‐TS is a key enzyme in the *de novo* pathway for the synthesis of folate by the parasite, *Pf*LDH catalysis, the final step in the generation of ATP by the parasite, and FP‐2 and plasmepsin II are essential hemoglobinase enzymes found in the acid food vacuole of the parasite that digests hemoglobin to release amino acids crucial for the growth and replication of the parasite. *Pf*DHODH and *Pf*HGXPRT are important enzymes in the *de novo* biosynthesis of pyrimidines and purine salvage pathway, respectively.

##### 2.6.1.1. Preparation of Target Proteins

The pdb file of the x‐ray crystalline structure of the proteins (*Pf*DHFR‐TS, PfLDH, FP‐2, PMS‐II, *Pf*DHODH, and *Pf*HGXPRT) was downloaded from the protein data bank at https://www.rcsb.org/ using their PDB IDs. The AutoDockTools was used in processing the proteins for docking. Despite some of the proteins having multiple chains, only Chain A of each protein was processed for docking. Water molecules were deleted from the proteins and the native ligands, and cofactors of the proteins were removed from their binding pockets. Polar hydrogen atoms were added to the proteins and the energy of the proteins minimized by the addition of Kollman charges [24, 25]. The processed proteins were saved in pdbqt format to be used for subsequent docking.

##### 2.6.1.2. Preparation of Ligands

The SMILES of the structures of the ligands (MAL, pyrimethamine, chloroquine, compound E64, compound EH58) were obtained from the PubChem online database and converted to pdb files (3D structures) using the OPENBABEL—chemical file format converter online tool (https://www.cheminfo.org/Chemistry/Cheminformattics/FormatConverter/index.html). AutoDockTools was utilized in adding hydrogen atoms and Gasteiger charges to the ligands [26]. The processed ligands (MAL, pyrimethamine, chloroquine, compound E64, compound EH58, compound DMS705, and hypoxanthine) were then saved in the pdbqt format for docking.

##### 2.6.1.3. Docking

AutoDock 4 was used to investigate the interaction of MAL with the active pockets of the target proteins at the atomic level [26]. The analysis employed Lamarkian genetic algorithm and default settings for the docking of a flexible ligand to a rigid protein. The binding pocket was defined by determining the co‐ordinate of the center of the native ligand using Microsoft Excel and adopting a grid cubic dimension of 60 × 60 × 60 Å about the center of the cocrystalized ligand to cover the entire active pocket [27, 28]. In docking involving *Pf*LDH, the ligands were docked against both the active pocket (oxamate binding site) and cofactor pocket (NADH binding site). The rest of the proteins were docked only at their main active pocket. Two set of 50 independent runs were done, and the protein–ligand complex of the conformer with the lowest estimated free energy of binding was selected and pdb file was written for further analysis of their binding characteristics. The protein ligand interaction profiler online tool (https://plip-tool.biotec.tu-dresden.de/plip-web/plip/index) was used to determine the type of interaction (hydrogen bond and hydrophobic interactions) between the ligands and key amino acid residues of the active pocket. The 3D graphical representations of the docked protein–ligand complexes were visualized using the PyMOL software, and the LigPlot^+^ Version v.2.3.1 was employed for the 2D graphical representations of the docked complex [29, 30]. To validate the docking protocol used and for comparison, reference ligand (pyrimethamine against DHFR‐TS, chloroquine against *Pf*LDH, compound E64 against FP‐2, EH58 against plasmepsin II, hypoxanthine against *Pf*HGXPRT, and compound DSM705 against *Pf*DHODH) is redocked against their respective proteins. Similarities between the docking characteristics of these reference ligand and their docking fingerprints reported in the literature were used to validate the docking procedure. The docking procedure was further validated using MD simulation.

#### 2.6.2. *In Silico* ADMET and Drug‐Likeness Prediction

Rule‐based filters such as Lipinski, Ghose, Veber, and Abbott bioavailability criteria were used to predict the likeness of MAL as a drug. SwissADME online tool (https://www.swissadme.ch/) and pkCSM online tool (https://biosig.lab.uq.edu.au/pkcsm/) were used to predict in silico ADMET properties and additional drug‐like properties of MAL [31]. ART was also assayed for comparison.

#### 2.6.3. MD Simulations

MD simulations were performed to determine the stability of all the docked MAL/protein complexes: MAL/DHFR‐TS (MAL‐4DP3), MAL/*Pf*LDH (MAL‐1LDG), MAL/FP‐2 (MAL‐3BPF), MAL/plasmepsin II (MAL‐1LF3), MAL/*Pf*HGXPRT (MAL‐30ZF), and MAL/*Pf*DHODH (MAL‐7K24) under conditions that mimic closely the physiological environment. The simulation gives insights into the stability of MAL at the active pocket of these target proteins, which is very crucial for understanding the inhibitory potential of the compound against these proteins. The pdb file of the docked MAL–protein complexes was uploaded unto the IMODS server (https://imods.iqfr.csic.es/) and the CABS‐Flex 3.0 server (https://lcbio.pl/cabsflex3/) for running MD simulations. The IMODS server, which uses normal mode analysis (NMA), was used to evaluate the motion stiffness, flexibility, and deformability of the docked protein–ligand complexes, while the CABS‐Flex 3.0 server was utilized in determining the root‐mean‐square fluctuations (RMSFs) of the complexes compared to the proteins to give further information on the stability of the protein–ligand complexes especially at the active pocket [32–35].

### 2.7. The Antifolate Potential of MAL Using Modified In Vitro Antiplasmodial Assay

To determine the interference of folate metabolism of *P. falciparum* parasites by MAL, a modified method by Petersen, E., 1987, was employed [36]. The protocol used was based on the hypothesis that the folic acid supplementation of culture media could decrease the antiplasmodial efficacy of agents that depend heavily on their ability to interfere with folate metabolism to kill the plasmodium parasites. Three in vitro experimental scenarios were created: (1) exposure of the trophozoites of 3D7 *P. falciparum* to MAL in a routine parasite culture media [21], (2) exposure of the trophozoites of 3D7 *P. falciparum* to MAL in a modified culture media supplemented with 2 mg/L folic acid, and (3) exposure of the trophozoites of 3D7 *P. falciparum* to MAL under a modified culture media supplemented with 4 mg/L folic acid [36]. All other assay conditions for running a routine antiplasmodial assay were maintained [21]. The parasites were subcultured at least twice in the modified culture media for the adaptation of the parasites to the media prior to the assay [36]. MAL at concentrations ranging from 100 to 0.39 μg/mL was used for the assays. A 32.58 μg/mL solution of sulfadoxine/pyrimethamine (SP) (20:1) was prepared and serially diluted 10‐fold and plated alongside MAL to validate the protocols used. The SYBR Green assay described already was used to quantify the antiplasmodial activity of the compound and SP in this assay.

### 2.8. Statistical Analysis

The IC_50_ of MAL and reference drugs was determined using Version 8.0 of GraphPad Prism software. The selectivity index (SI) of MAL against the trophozoites, schizonts, and gametocytes of both 3D7 and Dd2 lab strains of *P. falciparum* was calculated by finding the ratio of the CC_50_ of MAL against HepG2 liver cells to each of the IC_50_ of MAL against the different life stages of the two lab strains of *P. falciparum*. The IC_50_ of MAL and the standard compounds were presented as mean ± SD. All statistical analysis was by one‐way ANOVA followed by the Bonferroni multiple comparison test. Statistical significance was set at a *p* value of < 0.05.

## 3. Results

### 3.1. Cytotoxic Effect of MAL Against HepG2 Liver Cells

**FIGURE 1:**
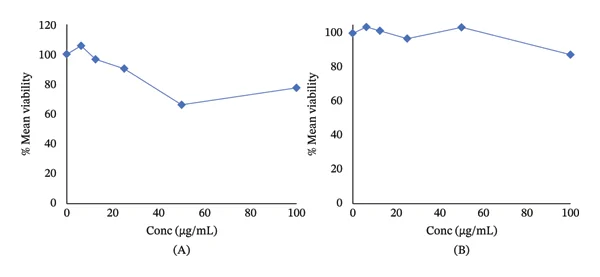
The effects of methylangolensate (A) and artesunate (B) against HepG2 liver cells.

MAL demonstrated minimal cytotoxic effects against HepG2 liver cells. The median cytotoxic concentration (CC_50_) of MAL (89.39 μg/mL or 189.95 μM) was similar to that of ART (99.01 μg/mL or 257.57 μM). An increase in concentration above the 20 μg/mL mark showed no drastic drop in %viability of the cells for both MAL and ART (Table 1 and Figure 1).

**TABLE 1:** The cytotoxic effects against HepG2 cells and the antiplasmodial effects of methylangolensate compared to artesunate against both 3D7 and Dd2 *P. falciparum*.

Compounds	CC_50_ values *μ*g/mL (μM)	Parasites strain	IC_50_ values *μ*g/mL (μM)	SI values (CC_50_/IC_50_)
MAL	89.39 (189.95)	3D7 Trophozoites	0.86 ± 0.1910 (1.83)	> 100
ART	99.01 (257.57)		0.00019 ± 0.0000197 (0.00049)	> 100

MAL	89.39 (189.95)	Schizonts	1.39 ± 0.3250 (2.95)	64.31
ART	99.01 (257.57)		0.0018 ± 0.000013 (0.0047)	> 100

MAL	89.39 (189.95)	Gametocytes	12.08 ± 2.2900 (25.69)	7.40
ART	99.01 (257.57)		0.005063 ± 0.000023 (0.013)	> 100

MAL	89.39 (189.95)	Dd2 Trophozoites	1.20 ± 0.4634 (2.55)	74.49
ART	99.01 (257.57)		0.00004246 ± 0.000023 (0.00011)	> 100

MAL	89.39 (189.95)	Schizonts	5.12 ± 3.8390 (10.88)	17.45
ART	99.01 (257.57)		0.00179 ± 0.000013 (0.0047)	> 100

MAL	89.39 (189.95)	Gametocytes	18.82 ± 3.5400 (39.99)	4.75
ART	99.01 (257.57)		0.0051 ± 0.000023 (0.013)	> 100

*Note:* Methylangolensate (MAL) and artesunate (ART). IC_50_s expressed as mean ± SD.

### 3.2. The Multistage Antiplasmodial Activity of MAL Against 3D7 Lab Strain *P. falciparum*

**FIGURE 2:**
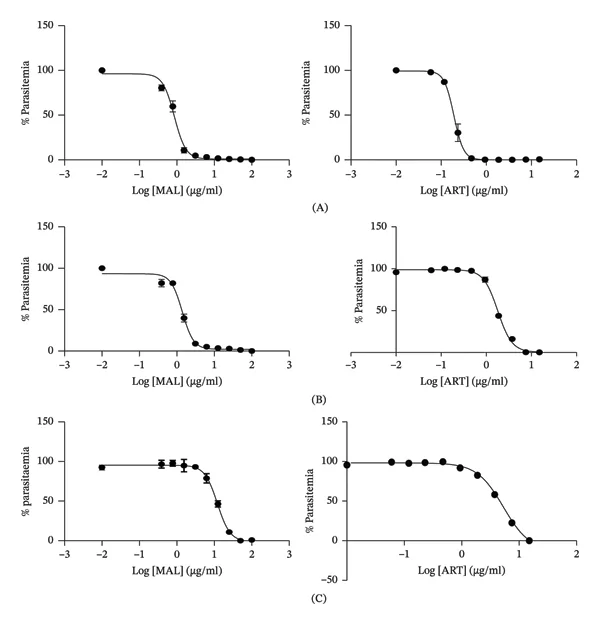
The effects of methylangolensate (MAL) and artesunate (ART) against (A) trophozoites, (B) schizonts, and (C) gametocytes of 3D7 lab strain *P. falciparum*.

The effects of MAL against the different stages of 3D7 *P. falciparum* are summarized in Table 1 and Figure 2. MAL demonstrated a good antiplasmodial effect against the trophozoites and schizonts of 3D7 *P. falciparum* (IC_50_ of 0.8568 μg/mL or 1.83 μM and 1.3900 μg/mL or 2.95 μM, respectively) (Table 1). MAL also demonstrated a moderate antiplasmodial activity against the gametocytes of 3D7 *P. falciparum* (IC_50_ of 12.0800 μg/mL or 25.69 μM). MAL was identified to be selectively toxic against the trophozoites and schizonts of 3D7 *P. falciparum* relative to HepG2 liver cells (SI above 100 and 64.31, respectively). The SI of MAL against the gametocytes of 3D7 *P. falciparum* relative to HepG2 cells was 7.40.

### 3.3. The Multistage Antiplasmodial Activity of MAL Against Dd2 Lab Strain *P. falciparum*

**FIGURE 3:**
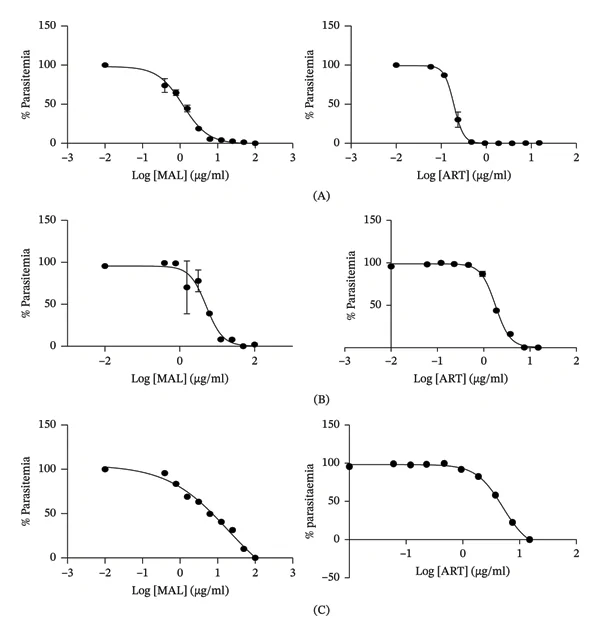
The effects of methylangolensate (MAL) and artesunate (ART) against (A) trophozoites, (B) schizonts, and (C) gametocytes of Dd2 lab strain *P. falciparum*.

The effects of MAL against the different stages of Dd2 *P. falciparum* are summarized in Figure 3 and Table 1. MAL demonstrated a good antiplasmodial effect against the trophozoites and schizonts of Dd2 *P. falciparum* (IC_50_ of 1.1960 μg/mL or 2.55 μM and 5.1210 μg/mL or 10.88 μM, respectively). MAL also demonstrated a moderate antiplasmodial activity against the gametocytes of Dd2 *P. falciparum* (IC_50_ of 18.8200 μg/mL or 39.99 μM). MAL was identified to be selectively toxic against the trophozoites and schizonts of Dd2 *P. falciparum* relative to HepG2 liver cells. The SI of MAL against the gametocytes of 3D7 *P. falciparum* relative to HepG2 cells was 4.75.

### 3.4. *In Silico* ADMET and Drug Likeness Prediction

**FIGURE 4:**
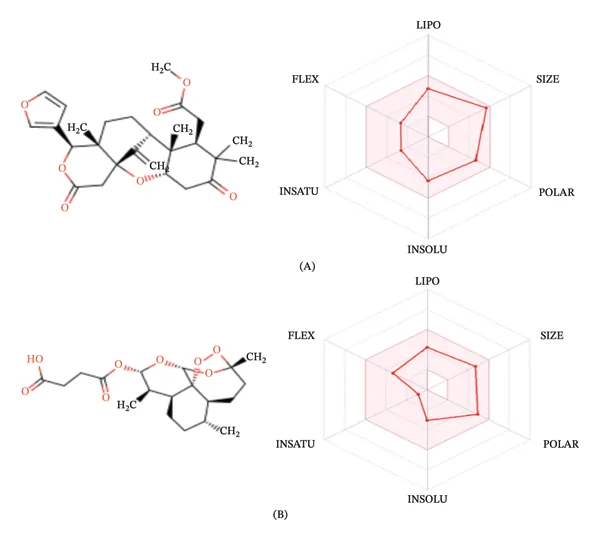
The bioavailability radar of methylangolensate (A) and artesunate (B) generated by SwissADME online server. Flexibility (FLEX), lipophilicity (LIPO), solubility (INSOLU), and saturation (INSATU). The pink zone represents acceptable bioavailability regions, and the red diagram represents the bioavailability parameters of the compounds. The red diagram completely within the pink zone represents the desirable bioavailability profile.

**FIGURE 5:**
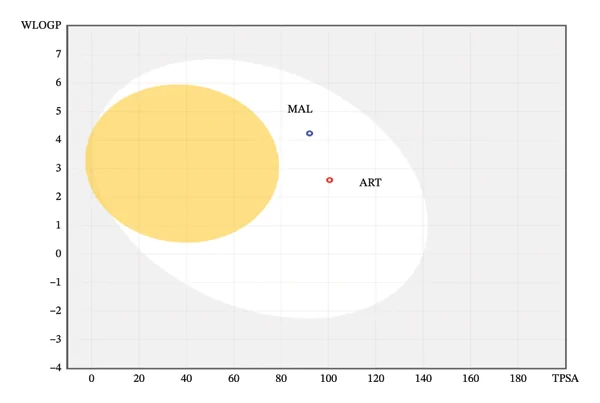
BOILED‐Egg model in SwissADME for predicting passive absorption. The yellow region indicates good CNS penetration; the white region represents high intestinal absorption; gray region represents poor intestinal absorption. Methylangolensate (MAL) (blue dot in the white region) and artesunate (ART) (red dot in the white region).

MAL met all the criteria set by Lipinski, Ghose, Veber, and Abbott bioavailability score for a drug‐like compound (Table 2). The bioavailability radar of MAL and ART generated by the SwissADME online tool was both within the pink zone, which indicates high intestinal absorption (HIA) (Figure 4). The BOILED‐Egg model from the SwissADME tool also assigned both MAL and ART to the white zone area, which represents the compounds with favorable oral absorption (Figure 5). According to the in silico ADMET prediction tool, MAL is likely to be completely absorbed upon oral administration and may have moderate CNS and BBB penetration. The limonoid is likely to be metabolized by CYP3A4 but less likely to be metabolized by CYP1A2 and may demonstrate approximately 28% total body clearance. MAL demonstrated no features pointing to drug toxicity. The in silico ADMET properties of MAL compared to ART are summarized in Table 3.

**TABLE 2:** Drug likeness prediction of methylangolensate based on Lipinski, Ghose, Veber, and Abbott bioavailability score (F).

Compounds	Lipinski	Ghose	Veber	*F*
Methylangolensate	Yes	Yes	Yes	0.55

**TABLE 3:** The ADMET properties of methylangolensate (MAL) compared to artesunate (ART).

Properties	Model name	Predicted value		Units
		MAL	ART	
Absorption	Water solubility	−5.452	−3.097	Log mol/L
Absorption	Caco‐2 permeability	0.924	0.863	LogPapp in 10^−6^ cm/s)
Absorption	Intestinal absorption	100	72.19	% Absorbed
Absorption	P‐glycoprotein substrate	No	Yes	Yes/No
Distribution	VDss (human)	−0.019	0.172	Log L/kg
Distribution	BBB penetration	−0.699	−0.954	Log BB
Distribution	CNS penetration	−2.71	−3.039	Log PS
Metabolism	CYP2D6 substrate	No	No	Yes/No
Metabolism	CYP3A4 substrate	Yes	Yes	Yes/No
Metabolism	CYP1A2 inhibitor	No	No	Yes/No
Metabolism	CYP2C19 inhibitor	No	No	Yes/No
Metabolism	CYP2C9 substrate	No	No	Yes/No
Metabolism	CYP2D6 inhibitor	No	No	Yes/No
Metabolism	CYP3A4 inhibitor	Yes	No	Yes/No
Excretion	Total clearance	0.283	0.969	
Excretion	Renal OCT2 substrate	No	No	Yes/No
Toxicity	AMES toxicity	No	No	Yes/No
Toxicity	Hepatotoxicity	No	No	Yes/No
Toxicity	Skin toxicity	No	No	Yes/No

### 3.5. Molecular Docking

#### 3.5.1. Docking of MAL Against DHFR‐TS

**FIGURE 6:**
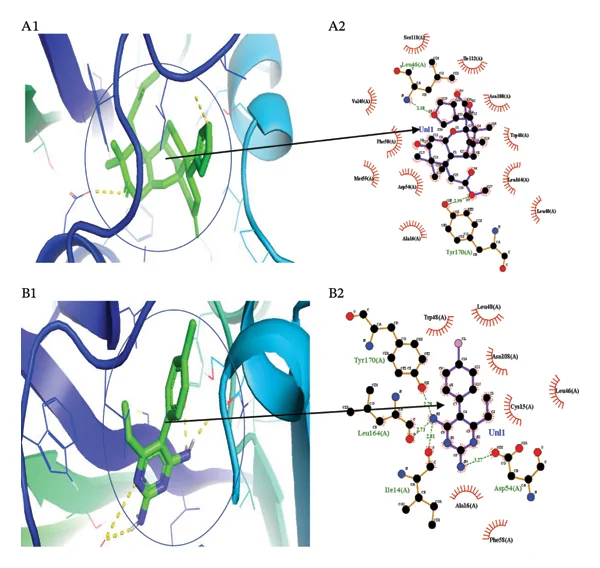
The 3D docked protein–ligand complex with corresponding LigPlot; methylangolensate (A1–A2) and pyrimethamine (B1–B2) against dihydrofolate reductase‐thymidylate synthase.

MAL was docked against the dihydrofolate (DHF) binding pocket of DHFR‐TS to elucidate the tendency of the compound to interfere with folate metabolism, which is crucial for the growth and replication of the *P. falciparum* parasites. To verify the inhibitory potential of the compound against DHFR‐TS, pyrimethamine was docked against the target protein to serve as a reference drug. MAL showed a stronger affinity (binding energy of −10.54 Kcal/mol) for the DHF binding pocket of DHFR‐TS than pyrimethamine (binding energy of −7.29 Kcal/mol). MAL interacted with some key amino acid residues through both hydrogen bond (ASP 54) and hydrophobic interactions (ALA 16, LEU 46, TRP 48, PHE 58, ILE 112, and LEU 164) (Table 4 and Figure 6) [37]. MAL formed hydrogen bond with ASP 54, which is known to be an important hydrogen bond acceptor for potent inhibitors like compound P218 [37–39]. MAL also showed hydrophobic interaction with one of the quadruple mutated sites (L164) of the target protein, which is an important interaction for inhibitors such as P218, which evade enzyme resistance [39, 40]. MAL also interacted with some key amino acid residues (PHE 58, ALA 16, and TRP 48) that are known to interact with ligands and inhibitors of the catalytic site of DHFR‐TS [39]. The strong affinity of MAL to the DHF binding pocket of DHFR‐TS and the interaction of the compound with key amino acid residues especially ASP 54 and LEU 164 strongly predicts MAL as a potential inhibitor of the DHFR‐TS enzyme.

**TABLE 4:** Molecular docking of methylangolensate and reference drugs or compounds against target proteins.

Protein–ligand complex	Binding energies (kcal/mol)	Hydrophobic interaction	Hydrogen bond interaction
MAL–4DP3	−10.54	ALA 16, LEU 46, TRP 48, PHE 58, ILE 112, LEU 164	ASP 54

PTM–4DP3	−7.29	ALA 16, LEU 40, LEU 46, TRP 48, TYR 170	ILE 14, CYS 15
			salt‐bridge (ASP 54)

MAL–1LDG‐1	−7.25	TYR 174, ALA 249	ALA 249
			salt‐bridge (ARG 171)

CRQ–1LDG‐1	−6.68	ILE 31, THR 101, LEU 112, THR 139, TYR 247	ILE 31, ASN 140, SER 245
			Halogen bond (ARG 171)

MAL–1LDG‐2	−8.05	ILE 31, THR 101	GLY 29, GLY 32, GLY 99, THR 101

CRQ–1LDG‐2	−7.20	VAL 26, ILE 31, PHE 52, THR 101, ILE 119	MET 30, ILE 31, THR 97
			salt‐bridge (ASP 53)

MAL–3BPF	−7.77	LEU 84, LEU 172, ALA 175, ASP 234	GLY 83

E64–3BPF	−5.14	TRP 43, ILE 85, ALA 175	GLY 83, ILE 85, ASN 86, ASN 173

MAL–1LF3	−7.39	VAL 78, THR 114, THR 217, ILE 290	SER 79, SER 218, ALA 219

EH58–1LF3	−10.76	ILE 14, ILE 32, TYR 77, VAL 78, PHE 111, PHE 120, ILE 123, TYR 192, ILE212	VAL 78, SER 79, TYR 192, GLY 216, SER 218

MAL–7KZ4	−9.41	TYR 168, LEU 172, LEU 187, PHE 188, LEU 191	LEU 172, ARG 265

DSM705–7KZ4	−9.22	PHE 188, ILE 263, ARG 265, VAL 532	HIS 185, ARG 265, TYR 528

MAL–3OFZ	−8.02	VAL 113, LYS 114, TYR 116, ILE 146, THR 152	LYS 77, ASP 148

HPX–3OFZ	−5.46	—	ASP 148, THR 149, GLY 150, LYS 151, THR 152, LEU 153

*Note:* Methylangolensate (MAL); pyrimethamine (PTM); chloroquine (CRQ); compound E64 (E64); compound EH58 (EH58); dihydrofolate reductase‐thymidylate synthase (4DP3); *P. falciparum* lactate dehydrogenase (1LDG‐1 main pocket, 1LDG‐2 cofactor pocket); falcipain II (3BPF); plasmepsin II (1LF3); *P. falciparum* dihydroorotate dehydrogenase (7KZ4); compound DSM705 (DSM705); *Plasmodium falciparum* hypoxanthine–guanine–xanthine phosphoribosyltransferase (3OFZ); hypoxanthine (HPX).

#### 3.5.2. Docking of MAL Against *Pf*LDH

**FIGURE 7:**
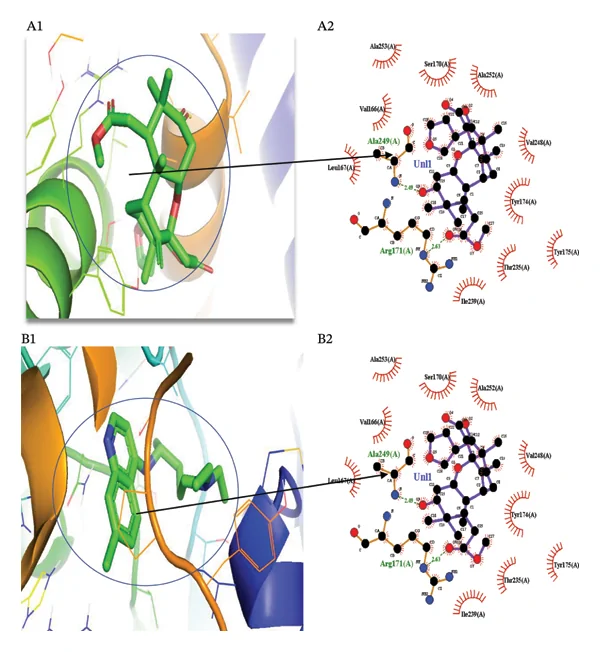
The 3D docked protein–ligand complex with corresponding LigPlot; methylangolensate (A1–A2) and chloroquine (B1–B2) against the oxamate binding pocket of *P. falciparum* lactate dehydrogenase.

**FIGURE 8:**
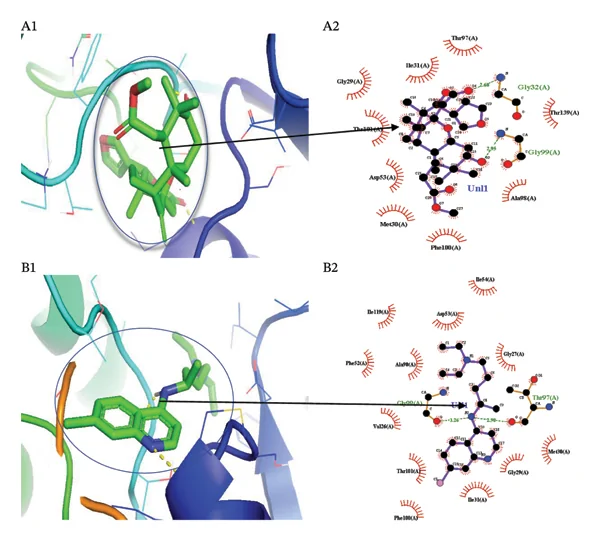
The 3D docked protein–ligand complex with corresponding LigPlot; methylangolensate (A1–A2) and chloroquine (B1–B2) against the cofactor binding pocket of *P. falciparum* lactate dehydrogenase.

*Pf*LDH has two major pocket (main active pocket‐1LDG1 and the cofactor binding site‐1LDG2) that interact with potential inhibitors. The main active site is inhibited by agents such as oxamate, while the cofactor binding site interacts with inhibitors such as chloroquine [41]. MAL was docked against both pockets of this important enzyme of the energy metabolic pathway of the parasite. Chloroquine was also docked against both pockets for comparison. MAL showed better affinity for both binding pocket (−7.25 and −8.05 Kcal/mol for 1LDG1 and 1LDG2, respectively) than chloroquine (−6.68 and −7.20 Kcal/mol for 1LDG1 and 1LDG2, respectively). MAL also demonstrated a stronger affinity for 1LDG2 than 1LDG1 similar to chloroquine [42]. MAL is likely to be a competitive inhibitor of the cofactor binding site of *Pf*LDH just as chloroquine. MAL formed four (4) hydrogen bonds (GLY 29, GLY 32, GLY 99, THR 101), while chloroquine formed three (3) hydrogen bonds (MET 30, ILE 31, THR 97) with key residues of the cofactor binding pocket of the enzyme [24, 42]. MAL formed hydrogen bonds with GLY 99, which is noted for interacting with potent inhibitors (including chloroquine) of the cofactor active site. MAL showed similar interaction (hydrophobic interaction) as chloroquine with ILE 31 and THR 101 residues of the binding pocket. The interaction of MAL with 1LDG‐1 is represented in Table 4 and Figure 7. Also, the interaction of MAL with 1LDG‐2 is represented in Table 4 and Figure 8.

#### 3.5.3. Docking of MAL Against Falcipain 2 (FP‐2)

**FIGURE 9:**
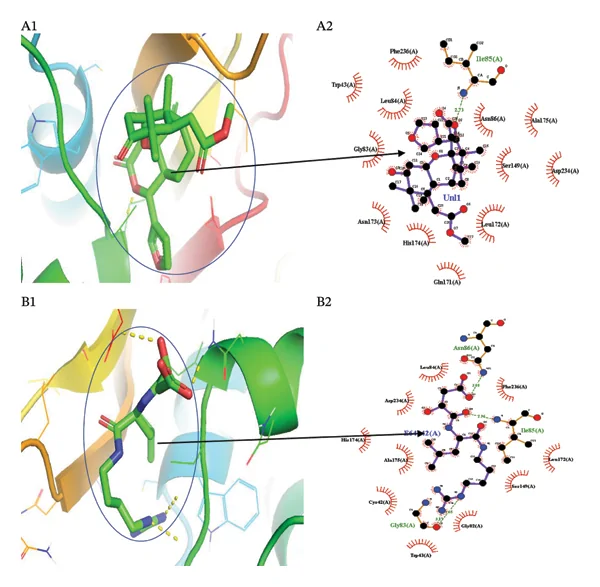
The 3D docked protein–ligand complex with corresponding LigPlot; methylangolensate (A1–A2) and compound E64 (B1–B2) against falcipain II.

MAL was docked against FP‐2 to investigate the ability of the compound to prevent the breakdown of hemoglobin in the acid food vacuole to deny the parasites of the necessary nutrition for growth and replication. Compound E64, which is the native inhibitor of the pdb structure of the enzyme, was redocked for comparison. MAL demonstrated a stronger affinity (−7.77 Kcal/mol) for the active pocket of the enzyme than compound E64 (−5.14 Kcal/mol). MAL formed a single hydrogen bond interaction with GLY 83, which also interacted with compound E64 through hydrogen bonding. GLY 83 is reported to be a key contributor to hydrogen bonding of strong inhibitors of the protein [39]. MAL also interacted through hydrophobic interaction with LEU 84 and ASP 234, which are known residues involved in key hydrophobic interactions with inhibitors (Table 4 and Figure 9) [43]. The better binding affinity of MAL for FP‐2 compared to E64 and the key interactions of the compound with crucial amino acids residues at the active pocket of the enzyme makes MAL and by extension its structural analogs promising inhibitors of the enzyme.

#### 3.5.4. Docking of MAL Against Plasmepsin II

**FIGURE 10:**
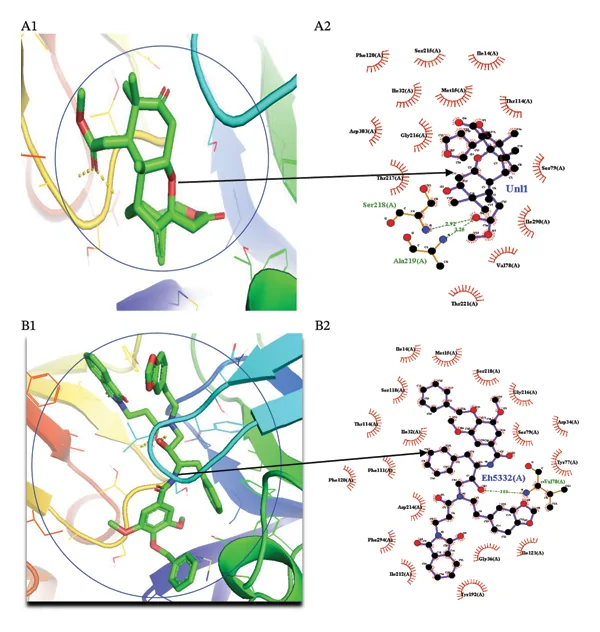
The 3D docked protein–ligand complex with corresponding LigPlot; methylangolensate (A1–A2) and compound EH58 (B1–B2) against plasmepsin II.

Plasmepsin II is also a hemoglobinase in the acid food vacuole of the plasmodium parasites. MAL was docked against the enzyme and its docking parameters compared to compound EH58, which is the native inhibitor of the pdb structure of the enzyme used. MAL showed slightly a lower affinity (−7.39 Kcal/mol) for the catalytic pocket of the enzyme compared to EH58 (−10.76 Kcal/mol). MAL formed three (3) hydrogen bonding interaction (SER 79, SER 218, ALA 219), while compound EH58 formed five (5) hydrogen bonds (VAL 78, SER 79, TYR 192, GLY 216, SER 218) with the active site residues. MAL did not interact with the catalytic dyad (ASP 34 and ASP 214) of the active pocket of the enzyme but formed strong hydrogen bonds with SER 218, which is in proximity with the dyad residues [44]. The compound also formed a hydrogen bond and hydrophobic bond with SER 79 and VAL 78, respectively, which are important flap residues that cover the binding pocket of the enzyme (Table 4 and Figure 10) [44]. Similarity in some interaction of MAL to EH58 and the important interaction of the compound with important residues such as SER 79, VAL 78, and SER 218 makes the compound a potential hemoglobinase inhibitor.

#### 3.5.5. Docking of MAL Against PfDHODH

**FIGURE 11:**
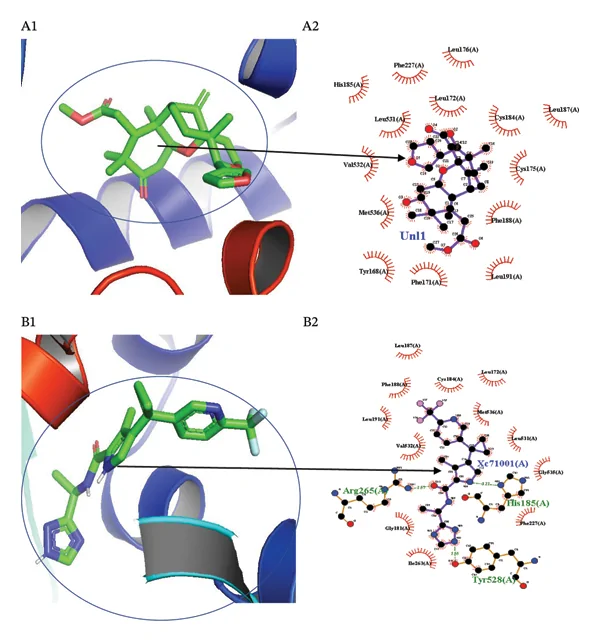
The 3D docked protein–ligand complex with corresponding LigPlot; methylangolensate (A1–A2) and DSM705 (B1–B2) against *P. falciparum* dihydroorotate dehydrogenase (*Pf*DHODH).

MAL was docked against *Pf*DHODH to predict the ability of the compound to inhibit the *de novo* biosynthesis of pyrimidines, which is crucial for the synthesis of the DNA of the parasites. MAL showed a slightly stronger affinity (binding energy, −9.41 Kcal/mol) for the active pocket of the enzyme compared to compound DSM705 (binding energy, −9.22 Kcal/mol) (Table 4 and Figure 11). MAL formed five hydrophobic interactions (TYR 168, LEU 172, LEU 187, PHE 188, and LEU 191) and two hydrogen bonds (LEU 172 and ARG 265), while DSM705 formed four hydrophobic interactions (PHE 188, ILE 263, ARG 265 and VAL 532) and three hydrogen bonds (HIS 185, ARG 265 and TYR 528) with the active site residues of the *Pf*DHODH (Table 4 and Figure 11). The active pocket of *Pf*DHODH is divided into two sections; one section is lined by HIS 185, ARG 265, and TYR 528, while the other section is a variable‐sized hydrophobic pocket consisting of residues such as TYR 171, PHE 188, TYR 168, and MET 536 [45]. MAL formed hydrogen bond with the key amino acid residue ARG 265 of the first pocket and two hydrophobic interactions (PHE 188, TYR 168) with residues of the other pocket. DSM705 interacted through hydrogen bonding with all the key residues of the first pocket (HIS 185, ARG 265, and TYR 528). Interaction of MAL with *Pf*DHODH generally mimics that of potential inhibitors of the enzyme.

#### 3.5.6. Docking of MAL Against PfHGXPRT

**FIGURE 12:**
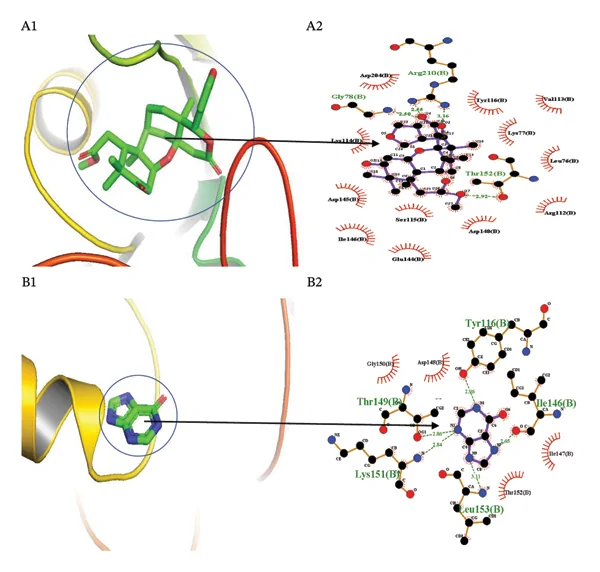
The 3D docked protein–ligand complex with corresponding LigPlot; methylangolensate (A1–A2) and hypoxanthine (HPX) (B1–B2) against *Plasmodium falciparum* hypoxanthine–guanine–xanthine phosphoribosyltransferase (*Pf*HGXPRT).

MAL was docked against *Pf*HGXPRT to ascertain whether the limonoid could block the salvage and subsequent utilization of purines in the synthesis of the DNA of the *P. falciparum* parasites. The limonoid was docked at the hypoxanthine binding site of the enzyme to see whether it could serve as a competitive inhibitor of the enzyme. MAL showed a higher affinity (−8.02 Kcal/mol) than hypoxanthine (−5.46 Kcal/mol) for the hypoxanthine binding site of *Pf*HGXPRT, which may predict competitive inhibition of the enzyme (Table 4). MAL formed a strong anchorage within the active site of the enzyme through both hydrophobic interactions (VAL 113, LYS 114, TYR 116, ILE 146, and THR 152) and hydrogen bond interactions (LYS 77 and ASP 148) with active site residues (Table 4 and Figure 12). Hypoxanthine formed only hydrogen bonds (ASP 148, THR 149, GLY 150, LYS 151, THR 152, and LEU 153) with the active site residues.

### 3.6. MD Simulation

#### 3.6.1. IMODS Dynamics

**FIGURE 13:**
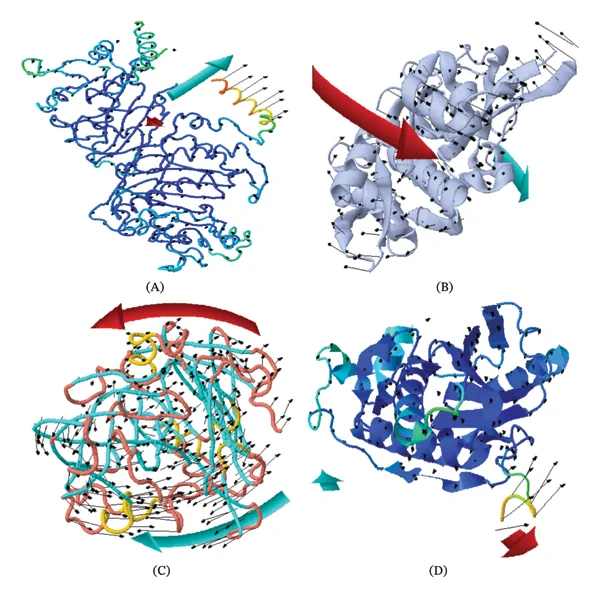
Molecular mobility and arrow field of docked methylangolensate‐4DP3 (A), methylangolensate‐3BPF (B), methylangolensate‐1LF3 (C), and methylangolensate‐3OZF (D) complexes. Molecular mobility is greater in the direction indicated by the longer affine arrow.

**FIGURE 14:**
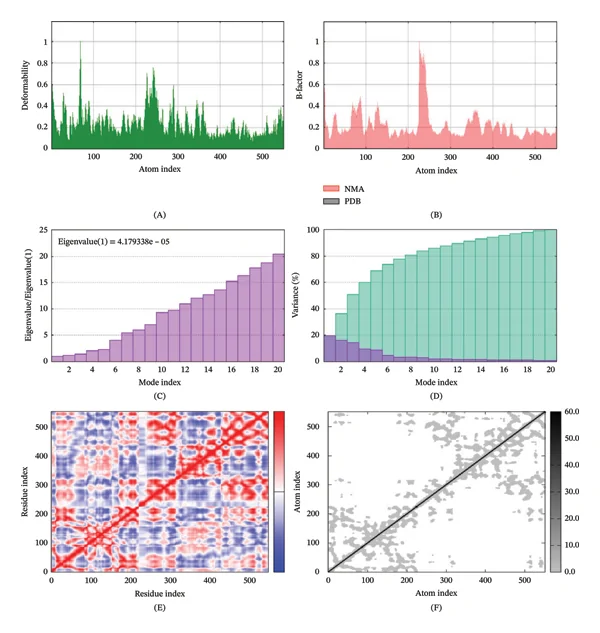
Molecular dynamics outputs from IMODS for docked methylangolensate–4DP3 complex: (A) Deformability graph, (B) B‐factor plots, (C) eigenvalue plots, (D) variance map plot, (E) correlation matrix plots, (F) elastic network model.

**FIGURE 15:**
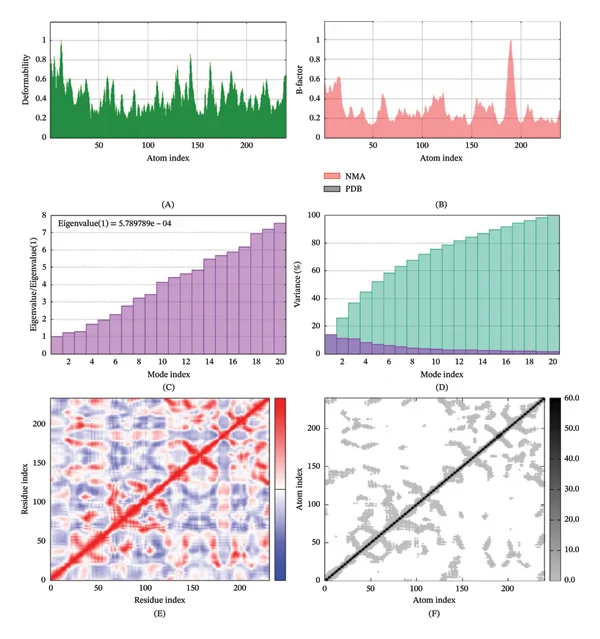
Molecular dynamics outputs from IMODS for docked methylangolensate–3BPF complex: (A) Deformability graph, (B) B‐factor plots, (C) eigenvalue plots, (D) variance map plot, (E) correlation matrix plots, (F) elastic network model.

**FIGURE 16:**
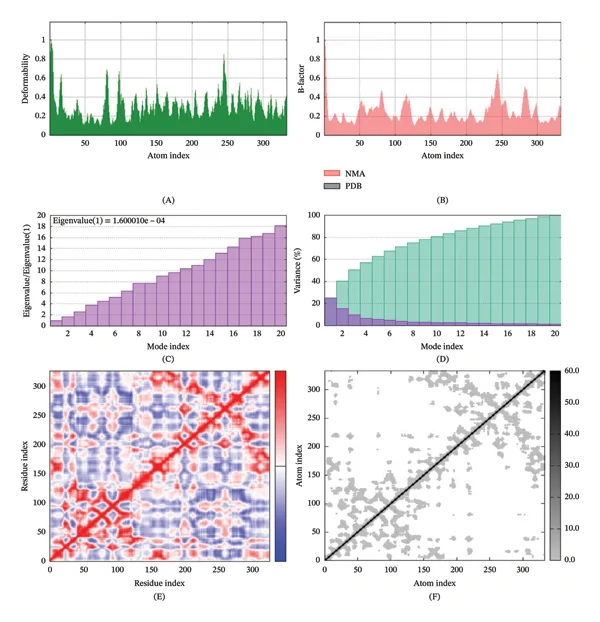
Molecular dynamics outputs from IMODS for docked methylangolensate–1LF3 complex: (A) Deformability graph, (B) B‐factor plots, (C) eigenvalue plots, (D) variance map plot, (E) correlation matrix plots, (F) elastic network model.

**FIGURE 17:**
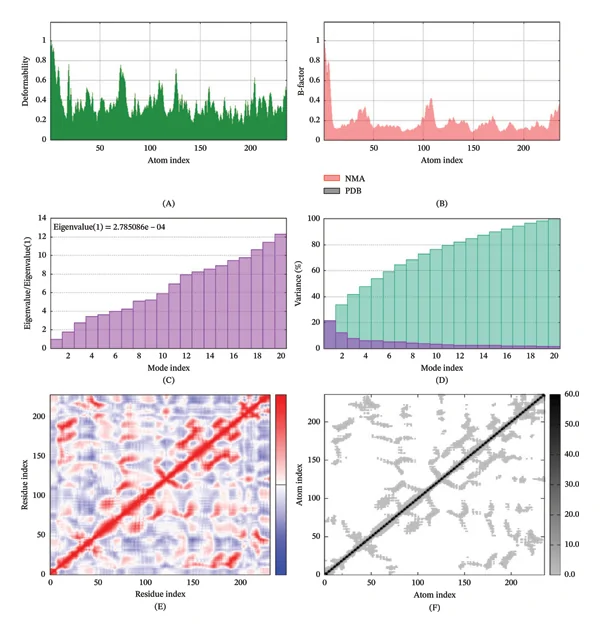
Molecular dynamics outputs from IMODS for docked methylangolensate–3OZF complex: (A) Deformability graph, (B) B‐factor plots, (C) eigenvalue plots, (D) variance map plot, (E) correlation matrix plots, (F) elastic network model.

The IMODS MD simulation was used to provide insight into the stability and molecular motion of the docked MAL‐protein complexes (Figure 13). The IMODS MD server generated a deformability plot, a B‐factor plot, eigenvalue, a variance map, a covariance map, and an elastic network for the docked MAL–protein complexes through NMA. The deformability plot and B‐factor plot show how the docked ligand influences the flexibility of the protein molecule. Higher peak areas indicate regions of the protein with high flexibility. The deformability potential of all the MAL–protein complex revealed minimal fluctuations, indicating a stable and strong interaction between MAL and the target proteins (Figures 14, 15, 16, 17). The eigenvalue is the measure of the motion stiffness of each normal mode, which is inversely related to the variance [32, 34, 46]. The lower the eigenvalue, the more flexible the protein and the higher the deformability of the protein. All the MAL–protein complex recorded very low eigenvalue (10^−5^–10^−4^), which signifies favorable interaction between MAL and the target proteins (Figures 14, 15, 16, 17). MAL–DHFR–TS complex demonstrated the lowest eigenvalue among the MAL–protein complexes. The covariance plot showed regions of correlation (red), anticorrelation (blue), and uncorrelation (white) within the target proteins. The covariance maps and elastic networks of the MAL–protein complexes were generally suggestive of stable complexes.

#### 3.6.2. CABS‐Flex Dynamics

**FIGURE 18:**
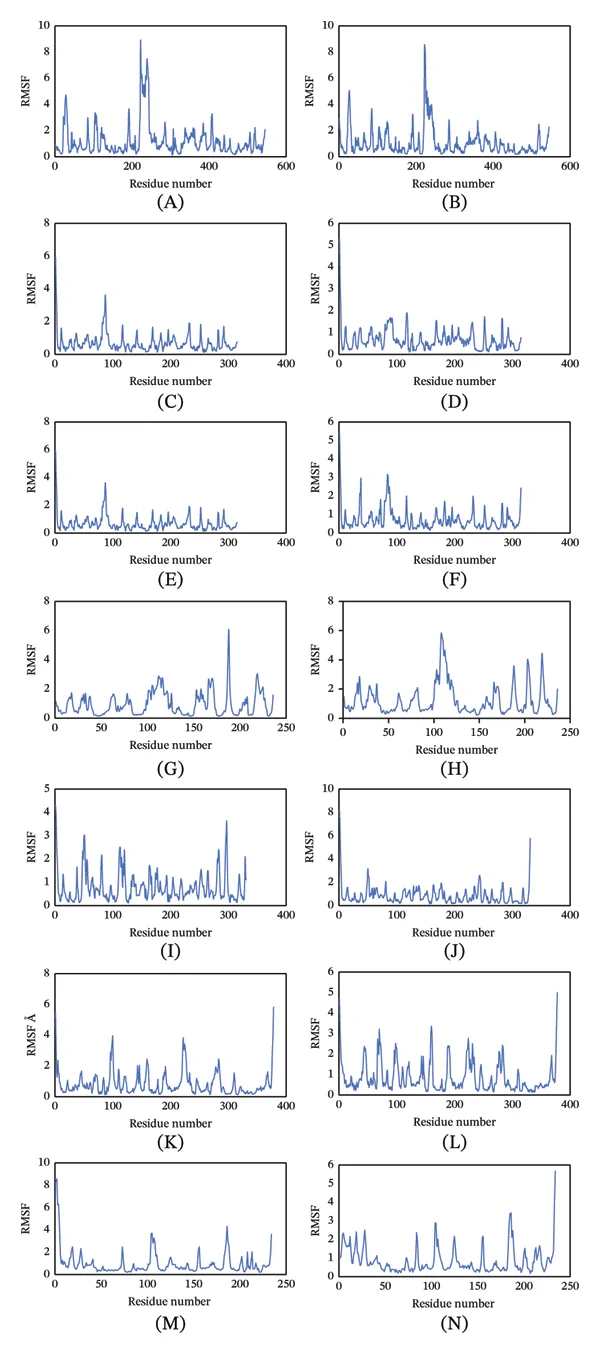
RMSF profile of proteins compared to protein–ligand complexes; (A) 4DP3 compared to (B) MAL–4DP3 complex; (C) 1LDG‐1 compared to (D) 1LDG–1‐MAL complex; (E) 1LDG‐2 compared to (F) 1LDG‐2–MAL complex; (G) 3BPF compared to (H) 3BPF–MAL complex; (I) 1LF3 compared to (J) 1LF3–MAL complex; (K) 7KZ4 compared to (L) MAL–7KZ4; (M) 3OZF compared to (N) 3OZF–MAL generated from the CABS‐Flex 3.0 online server [47].

The CABS‐Flex 3.0 online server was used to determine the average RMSF for the entire undocked protein structures compared to the docked MAL–protein complexes (Figure 18 and Table 5). Also, to estimate the effect of MAL on the active pockets of the target proteins, the average RMSF of the amino acid residues that interacted with MAL was compared to their average RMSF in the undocked protein (Figure 18 and Table 5). RMSF can identify and quantify local changes in the protein structure when a ligand interacts with specific residues of the protein. A RMSF < 2 Å is generally regarded as a sign of stability of the protein–ligand complex. The presence of MAL in the binding pocket of the target proteins had a minimal effect on the average RMSF of the entire structure of the undocked proteins (Figure 18 and Table 5). The average RMSF of the active site residues that interacted with MAL were similar for both docked and undocked proteins for all the target proteins. These findings further strengthen the claim made by the IMODS dynamics on the stability of all the MAL–protein complexes under dynamic conditions.

**TABLE 5:** Average RMSF of the fluctuation plot of protein compared to protein–ligand complex generated by Cabs‐Flex 3.0 online server.

Structure	Average RMSF (Å)	
	Entire structure	Active site residues
4DP3	1.064	0.496
MAL–4DP3	1.086	0.768
1LDG‐1	0.650	0.276
MAL–1LDG‐1	0.650	0.258
1LDG‐2	0.650	0.753
MAL–1LDG‐2	0.651	0.885
3BPF	1.249	1.162
MAL–3BPF	1.158	1.106
1LF3	0.819	1.036
MAL–1LF3	0.776	1.1780
3OZF	0.9775	0.5603
MAL–3OZF	0.8671	0.5930
7KZ4	0.8061	0.4757
MAL–7KZ4	0.8494	0.3555

*Note:* Methylangolensate (MAL); dihydrofolate reductase‐thymidylate synthase (4DP3); *P. falciparum* lactate dehydrogenase (1LDG‐1 main pocket, 1LDG‐2 cofactor pocket); falcipain II (3BPF); plasmepsin II (1LF3); *P. falciparum* dihydroorotate dehydrogenase (7KZ4); *Plasmodium falciparum* hypoxanthine–guanine–xanthine phosphoribosyltransferase (3OFZ).

### 3.7. The Antifolate Potential of MAL

The results of the effects of folic acid supplementation on the antiplasmodial effects of MAL and SP are summarized in Table 6. There was a slight decline in the antiplasmodial efficacy (increased IC_50_) of MAL with increasing concentration of folic acid in the culture media. This finding coupled with the potent interaction of MAL with DHFR‐TS predicted by the molecular docking and MD simulations may provide a strong basis for suggesting the inhibition of folate metabolism as part of the mechanism underlying the antiplasmodial effects of MAL. However, since the antiplasmodial activity of MAL was only slightly affected by folic acid supplementation of culture media, it may indicate that MAL could have other mechanism contributing to its antiplasmodial effects. The promising interaction of MAL with the other target proteins (*Pf*LDH, FP‐2, plasmepsin II, *Pf*DHODH and *Pf*HGXPRT) predicted by the in silico assays may provide some evidence to this claim. The antiplasmodial effects of SP also declined in the presence of folic acid supplementation, which was used to validate the protocols used.

**TABLE 6:** The effect of folic acid supplementation on the anti‐plasmodial efficacy of MAL in a modified in vitro anti‐plasmodial assay.

Compounds	IC_50_ values (*μ*g/mL)		
	NFM	FM‐2	FM‐4
MAL	0.9885 ± 0.0082	1.173 ± 0.1790 (ns)	1.2780 ± 0.9965 (ns)
SP	0.6180 ± 0.0066	1.2770 ± 0.0845 (ns)	ND

*Note:* Methylangolensate (MAL) and sulfadoxine/pyrimethamine (SP). No folic acid supplementation (NFM), folic acid supplementation at 2 mg/L (FM‐2) and folic acid supplementation at 4 mg/L (FM‐4). Not determined (ND). Statistical analysis was by one‐way ANOVA followed by the Bonferroni multiple comparison test, ns (not statistically significant) *p* value > 0.05 for MAL in FM‐2 (*p* value = 0.9925) and MAL in FM‐4 (*p* value = 0.9620) compared to MAL in NFM and for SP in FM‐2 (*p* value = 0.6275) compared to SP in NFM. Statistical significance was set at *p* value < 0.05.

## 4. Discussion

Bickii et al. were the first to report on the antiplasmodial effect of MAL [11]. The study isolated five limonoids from *Khaya grandifoliola* C.D.C. (Meliaceae) and identified them to be effective against chloroquine‐resistant W2/Indochina strain of *P. falciparum* (IC_50_, 1–10 μg/mL). The activity of the limonoids was gedunin (1.25 μg/mL) > 7‐deacetylkhivorin > MAL (5.39 μg/mL) > 1‐deacetylkhivorin > 6‐acetylswietenolide [11]. Prior to the study by Bickii et al., the antiplasmodial activity of limonoids (IC_50_) had been reported to range from 0.72 to 50 μg/mL with gedunin been the most potent [8, 48–51]. Bickii et al. also isolated MAL and some other limonoids from *E*. *angolense* (Welw.) C.DC. and reported MAL to have a lower antiplasmodial effect (IC_50_, 22.90 μg/mL) against the chloroquine‐resistant W2 strain compared to what was previously reported by Bickii et al. [11, 52]. A study by Sidjui et al. reported on a similar activity of MAL against chloroquine‐sensitive (*Pf*3D7) (5.66 μg/mL) and chloroquine‐resistant (*Pf*INDO) (5.62 μg/mL) strains of *P*. *falciparum*, which were similar to the activity of MAL reported by Bickii et al. against chloroquine‐resistant W2/Indochina strain of *P. falciparum* [11, 53]. Another study by Amang à Ngnoung et al., reported MAL to be less effective against the chloroquine‐sensitive 3D7 *P. falciparum* parasites compared to what was reported by Sidjui et al. (IC_50_ of 165.19 μg/mL against 5.66 μg/mL) [13]. The same study also identified MAL to be only moderately effective against the chloroquine‐resistant Dd2 strain of *P. falciparum* (IC_50_, 20.51 μg/mL). So far, the antiplasmodial effect of MAL reported in the literature ranges from 5.39 to 22.90 μg/mL against chloroquine‐resistant strains and 5.66–165.19 μg/mL against chloroquine‐sensitive strains.

There appears to be some slight variation in the literature regarding the antiplasmodial effects of MAL. Some studies have reported the limonoid (MAL) to be very active, and others have reported it to be only moderately active against *P. falciparum* parasites. This could be due to the differences in the strain of the *P. falciparum* parasites tested, the stage of the parasites (synchronized ring stage against unsynchronized asexual parasites) tested, assay protocol observed, and some interlaboratory differences. This study unlike previous studies tested the stage‐specific antiplasmodial effect of MAL and identified the limonoid to be very active against the trophozoites and schizonts (IC_50_, 0.86–5.12 μg/mL or 1.83–10.88 μM) but less active against the gametocytes (IC_50_, 12.08–18.82 μg/mL or 25.69–39.99 μM) of both 3D7 *P. falciparum* and Dd2 *P. falciparum* parasites. Since this current study tested the activity of MAL against different stages of the parasite, direct comparison with previous studies that tested the effect of MAL against unsynchronized parasites might be difficult. However, the antiplasmodial effect of MAL identified by this current study against the asexual stage parasites (synchronized trophozoites and schizonts) is consistent with some of the studies that reported on the antiplasmodial effect of MAL against unsynchronized asexual parasites. Actually, MAL demonstrated similar antiplasmodial activity against the chloroquine‐resistant strains and chloroquine‐sensitive strain (asexual stage), which was similar to what was reported by Sidjui et al. [53]. This pattern has also been reported for some limonoids, and it has actually been proposed that chloroquine resistance might have a minimal effect on the antiplasmodial activity of plant extracts that depends on limonoids for their antimalarial effects [54]. Generally, the antiplasmodial activity of MAL in this study is consistent with the activity of limonoids reported in the literature (0.72–50 μg/mL) [11].

The rank order of the activity of MAL against the respective stages of the plasmodium parasites was trophozoites > schizonts > gametocytes. Alpha onocerin, a triterpenoid derived from *Huperzia phlegmaria* also demonstrated a similar stage specific antiplasmodial effect as MAL (a tetranortriterpenoid) reported by this study [55]. Actually, artemisinin derivatives and nonartemisinin derivatives used in the treatment of malaria in the clinics have been reported to be very effective against the asexual stage of the *P. falciparum* parasites with little or no effect against the gametocytes [16, 18]. A loss in gametocyte activity for some compounds can be attributed to decreased metabolic activities in gametocytes. Chloroquine and artemisinin derivatives that are dependent on the rate of hemoglobin metabolism for their antiplasmodial activity turn to have reduced activity against the matured gametocytes which do not require hemoglobin metabolism for survival [56, 57]. The parasite surface anion channel (PSAC), which is responsible for the uptake of nutrients and some drugs in the asexual parasites, is absent in gametocytes. The membrane composition also varies between asexual parasites and gametocytes, with asexual parasites rich in phospholipids required for membrane biogenesis during replication. By contrast, the membranes of mature gametocytes show increased cholesterol levels (free and esterified) and sphingolipids consistent with increased membrane stability [56, 57]. These specific features of gametocytes make them less susceptible to many drugs and phytochemicals that have been reported to possess antiplasmodial effects.

Limonoids are polyoxygenated terpenoids, which are biosynthetically related to quassinoids and terpenes [8]. The antiplasmodial effects of terpenes and quassinoids are well established in the literature [8, 11]. Like the terpenes and quassinoids, the antiplasmodial effects of limonoids have been related to the presence of α, β‐unsaturated ketone in Ring A (C‐1 and C‐2) of their chemical structure [11]. The α, β‐unsaturated ketone in Ring A of these compounds has been proposed to be involved in the reaction with the DNA of the *P. falciparum* parasites in a Michael addition reaction, which leads to the death of the parasites [11, 53, 58]. Structural activity relation (SAR) of the ceramicines (a class of limonoid) also demonstrated the importance of the α, β‐unsaturated ketone in Ring A for antiplasmodial activity [59]. Ceramicine W, which was identified to be the most potent (IC_50_, 1.20 μM) among several ceramicines against the ring‐form‐synchronized parasites of 3D7 *P. falciparum*, possesses this α, β‐unsaturated ketone in its Ring A [59]. Also, gedunin, which has been reported to be the most potent limonoid in vitro against several strains of *P. falciparum* parasites, is known to benefit significantly from the presence of this α, β‐unsaturated ketone in its Ring A [11]. In fact, the reduction of the double bond in compounds possessing an α, β‐unsaturated carbonyl moiety at C1/C2 in Ring A has been shown to decrease the antimalarial activity [8, 11]. MAL, which is known to possess a β‐substituted furan ring, lactone ring, ketone group, cyclic ether linkage, and exocyclic methylene group, lacks the α, β‐unsaturated ketone in Ring A. Rather, MAL has a saturated cyclic ketone at C‐3, which result in the reduction of the antiplasmodial effect compared to limonoids like gedunin with the α, β‐unsaturated ketone in Ring A. Despite the absence of α, β‐unsaturated ketone in Ring A of MAL, the limonoid also possess certain structural features, which contributes to its antiplasmodial effects. The β‐substituted furan ring at C‐17 and the epoxide at C‐14 of MAL are known to be crucial for antiplasmodial activity of limonoids [53, 59]. Actually, the presence of the epoxide at C‐14 in conjunction with α, β‐unsaturated ketone in Ring A is known to produce superior antiplasmodial effects. In the SAR of the ceramicines, it was inferred that the double bond at C‐14 is essential for activity, the oxidation of the double bond to an epoxide like presence in MAL increases activity, but further oxidation into a ketone decreases activity [59]. The addition of the hydroxyl group at C‐6 of MAL to form 6‐methylhydroxyangolensate has been reported to significantly decrease the antiplasmodial activity of MAL (IC_50_ of MAL significantly decreased from 5.39 to 21.59 μg/mL following the hydroxyl substitution at C‐6) [11]. This suggests that the nature of substitution at C‐6 is critical for the antiplasmodial effects of derivatives of MAL. Just as MacKinnon, Durst and Arnason 1996 tested the SAR of gedunin and its derivatives, the promising antiplasmodial effect of MAL warrants the SAR of MAL and its derivatives [54]. Based on the SAR reported for limonoids, derivatives of MAL should be designed and synthesized and tested against *P. falciparum* parasites to determine SAR. This might lead to the discovery of important structural features of MAL and its derivatives that could guide a structure‐based drug design, which could yield very potent antimalarial agents. From the SAR reported for limonoids so far, structural modification should target Ring A (especially derivatives with α, β‐unsaturated carbonyl moiety at C1/C2), the β‐substituted furan ring, substitution at C‐6, and the opened Ring B (which has been reported to decrease activity). From the SAR of the ceramicines, the oxidation of the furan ring to a 2‐furanone may help form derivatives with increased antiplasmodial activity [59]. Also, the replacement of hydroxyl groups by acetoxy groups in Ring A and Ring B especially at C‐1 or C‐7 can be adopted in the development of MAL derivatives since this substitution has been reported to increase antiplasmodial effects in some meliacolide derivatives [7, 11].

In vitro cytotoxicity studies are useful for the screening of compounds and give important insights into compounds that are safe enough to proceed forward along the drug discovery pipeline. The ability of compounds to stop the proliferation of parasites without being hazardous is estimated using the SI. The SI is the ratio of cytotoxicity (CC_50_) on human cells to the antimalarial activity (IC_50_) of the tested compound or extract. A low SI indicates that the antiplasmodial effect of the compound might be due to the compound been toxic, whereas a high SI indicates that the compound might possess an intrinsic pharmacological effect against the parasites. A higher SI score is important for a safer course of treatment. Compounds are categorized as not cytotoxic if CC_50_ > 90 μg/mL, as moderately cytotoxic if it is between 2 and 89 μg/mL, and as cytotoxic if CC_50_ < 2 μg/mL [60]. MAL demonstrated weak cytotoxic effects (CC_50_ = 89.39 μg/mL) against HepG2 cells similar to ART (CC_50_ = 91.86 μg/mL). A study also reported on a weak cytotoxic effect (CC_50_ = 72.74 μg/mL) of MAL against RAW 264.7 cells [8]. Another study also reported on a very weak cytotoxic effect of MAL (CC_50_ > 200 μg/mL) against the HEK239T cell line [53]. MAL was selectively toxic against the asexual parasites (trophozoites and schizonts) but less selective against the gametocytes. This finding is consistent with the study by Sidjui et al., which identified MAL to be selectively toxic against the asexual parasites (unsynchronized) of both 3D7 and INDO strains of *P. falciparum* [53]. This suggests that MAL might possess intrinsic trophozoitocidal and schizontocidal activities, which is crucial for agents to be developed for the treatment of the clinical phase of malaria in humans.

MAL satisfied all the criteria set by Lipinski, Ghose, Veber, and Abbott for a compound with drug‐like features with no violations. The Swiss ADME tool also identified MAL to possess favorable ADMET properties, which is very vital in its development as a drug candidate. According to the tool, MAL is likely to be completely absorbed following oral administration, which could increase the bioavailability of the compound and guarantee the accumulation of the compound in its intraerythrocytic target (crucial for activity). This is important because, gedunin’s antiplasmodial activity decreased significantly when tested in vivo against *P. berghei* infection, which was attributed to issues pertaining to low bioavailability. The in silico study did not identify any feature of MAL that points to potential drug toxicity, which corroborate the findings from the in vitro cytotoxicity assay. Also, the findings of this study are similar to other studies that predicted the drug likeness of MAL using in silico techniques [19].

DNA replication is indispensable to the survival of the *P. falciparum* parasites. During the intraerythrocytic development cycle (IDC), *P. falciparum* undergoes asynchronous schizogony, requiring multiple rounds of DNA replication to produce merozoites, which are necessary for maintaining infection and transmitting to new red blood cells. To sustain this extensive DNA replication of the parasites depends on the constant supply of key metabolites, especially the purines and pyrimidines [61]. Hence, anything that hinders the persistent supply of these nitrogenous bases (essential component of nucleic acids) could halt the growth and replication of the *P. falciparum* parasites and eventually lead to death. *Pf*DHODH, *Pf*HGXPRT, and *Pf*DHFR‐TS are important enzymes in the synthesis of the DNA of the *P. falciparum* parasites. Interestingly, MAL demonstrated some of its best interactions in the docking studies against these enzymes. The antiplasmodial effects of MAL may strongly involve the inhibition of DNA replication of the parasites through impairing the function of multiple biochemical enzymes engaged in the generation of nucleotide bases necessary for the biosynthesis of parasitic nucleic acids. Mechanistically, MAL may block the *de novo* synthesis of pyrimidines through the inhibition of *Pf*DHODH, prevent the synthesis of purines by blocking the salvage of hypoxanthine into IMP by inhibiting *Pf*HGXPRT, and deprive the parasites of tetrahydrofolate (by inhibiting *Pf*DHFR‐TS), which is essential for both purine and pyrimidine biosynthesis of the parasite. Collectively, these predicted effects of MAL against these enzymes might lead to substantial parasitic death and eventual resolution of the clinical manifestation of malaria.

The growth of the trophozoites depends heavily on the digestion of host hemoglobin. The trophozoites utilizes both FP‐2 and PMS‐II to digest host hemoglobin into heme and globin, which are essential for the synthesis of parasitic proteins [61]. The 4‐aminoquinoline antimalarial drugs such as chloroquine are known to produce their antimalarial effects through the inhibition of the detoxification of heme to hemozoin. MAL showed a strong inhibitory potential against both FP‐2 and PMS‐II. The predicted inhibition potential of FP‐2 and PMS‐II by MAL might starve the parasite of the essential amino acids crucial for survival and might kill the parasites through the accumulation of undigested hemoglobin and its intermediates in the food vacuoles, causing intense cellular stress and destroying the metabolic balance of the parasites [61]. The potential inhibition of *Pf*LDH by MAL as predicted by the docking studies might also tamper with the survival of the parasites by denying their access to ATP. *Pf*LDH and human LDH have similar function, but their amino acid sequences are less similar [62]. Therefore, MAL may successfully kill the parasites through selective inhibition of *Pf*LDH with no effect on the human LDH. Interestingly, *Pf*LDH is found in all the five species of the plasmodium parasites, suggesting a potential broad‐spectrum effect of MAL against the parasites [62]. These biochemical enzymes used in the in silico studies are known to accumulate greatly in the trophozoites and in some cases the schizonts but not in the gametocytes [63]. This might be the reason why the activity of MAL was more pronounced against the asexual parasites (trophozoites and schizonts) than the sexual stage (gametocytes).

Despite the promising interaction of MAL with all the target proteins, it was identified that the interaction of MAL was strongest against *Pf*DHFR‐TS. This suggests that interfering with folate metabolism may be a major mechanism underlying the antiplasmodial effects of MAL. Gedunin (another limonoid) and its structural analogs have been predicted through docking studies to be potent inhibitors of *Pf*DHFR [64]. The predicted binding affinity of MAL (−10.54 Kcal/mol) against *Pf*DHFR from the docking studies is stronger than reported for gedunin and its derivatives (−7.5 to −9.5 Kcal/mol). As proposed by these computational studies, MAL might be a better antifolate agent than gedunin and its derivatives. In vitro target‐based assay against *Pf*DHFR‐TS is warranted for confirmation. To validate the tendency of MAL to interfere with folate metabolism, the effectiveness of MAL as an antiplasmodial agent was tested under varying concentration of folic acid–supplemented media. It was identified that increasing folic acid concentration decreased the antiplasmodial effects of the compound, which might validate the inhibition of folate metabolism as an important mechanism that contributes to the antiplasmodial effects of MAL. These probable antifolate effects of MAL could provide enough basis for investigating the application of the compound in the management of other diseases such as cancers, autoimmune diseases, and infectious disease, which benefit from antifolate therapy.

## 5. Conclusion

MAL isolated from the stem bark of *E*. *angolense* (Welw.) C.DC. has demonstrated great prospect as an important lead for the development of novel antimalarial agents. The limonoid (MAL) likely contributes to the folkloric use of *E*. *angolense* (Welw.) C.DC. in the treatment of malaria. The potential effects of MAL against the plasmodium parasites may involve multiple targets, which might affect folate metabolism, energy metabolism, DNA replication, and nutrition of the parasites. Interfering with folate metabolism may be a major mechanism involved in the antiplasmodial effects of MAL. However, since the molecular docking and MD studies are only predictive and not conclusive, it would be crucial that further studies like enzyme inhibition assays be done to verify these predicted mechanisms.

### 5.1. Limitations

The docking results remain predictive and require additional in vitro target‐based assays or other experimental validation to confirm the proposed molecular mechanism of MAL against the *P. falciparum* parasites. The study did not report on the in vivo antiplasmodial effect of MAL against *P. berghei* infection in mice. The potential of MAL to serve as a partner drug to conventional antimalarial agents in combination therapy was not investigated.

## Author Contributions

Kwesi Boadu Mensah, Charles Ansah, Isaac Kingsley Amponsah, and Arnold Donkor Forkuo developed the concept for the study; Jude Tetteh, Jemima Aggrey Appiah, and Felix Zoiku developed the methods for the study; Isaac Kingsley Amponsah and Bismark Aboagye‐Adjei were responsible for the isolation and characterization of the methylangolensate; Jude Tetteh and Felix Zoiku were responsible for the execution of the in vitro antiplasmodial assays; Jude Tetteh conducted the in silico studies including the molecular docking and MD studies; Jude Tetteh and Felix Zoiku analyzed the data generated; Jude Tetteh, Derwaa Keteku, and Felix Zoiku were responsible for the drafting, review, and editing of the manuscript under the supervision of Kwesi Boadu Mensah, Charles Ansah, Isaac Kingsley Amponsah, and Arnold Donkor Forkuo. All authors had made significant contributions to the research work.

## Funding

The team did not receive any external funding.

## Disclosure

The published version of the manuscript has been read and approved by all authors. All authors have given their consent to be accountable for the research presented.

## Ethics Statement

Every method was used in compliance with the applicable rules and regulations. The Institute of Research, Innovation and Development (IRID) of Kumasi Technical University gave its approval to all experimental procedures (Ref. No: IRID/EC2025/HS0084).

## Conflicts of Interest

The authors declare no conflicts of interest.

## Data Availability

This article contains all of the relevant data created or analyzed during the investigations.
